# Three dysregulated miRNAs control kallikrein 10 expression and cell proliferation in ovarian cancer

**DOI:** 10.1038/sj.bjc.6605634

**Published:** 2010-03-30

**Authors:** N M A White, T-F F Chow, S Mejia-Guerrero, M Diamandis, Y Rofael, H Faragalla, M Mankaruous, M Gabril, A Girgis, G M Yousef

**Affiliations:** 1 Department of Laboratory Medicine, and the Keenan Research Centre in the Li Ka Shing Knowledge Institute, St Michael's Hospital, Toronto, ON, Canada M5B 1W8; 2 Department of Laboratory Medicine and Pathobiology, University of Toronto, Toronto, ON, Canada M5G 1L5; 3 Department of Pathology, London Health Sciences Center and University of Western Ontario, London, ON, Canada N6A 5W9

**Keywords:** bioinformatics, ovarian cancer, kallikrein, KLK, miRNA, tumour markers

## Abstract

**Background:**

Kallikrein-related peptidases (KLKs) are a family of serine proteases that have been shown to be dysregulated in several malignancies including ovarian cancer. The control of kallikrein genes and their physiological function in cancer is not well understood. We hypothesized that microRNAs (miRNAs) represent a novel mechanism for post-transcriptional control of KLK expression in cancer.

**Methods:**

We first analysed miRNA expression in ovarian cancer *in silico*. A total of 98 miRNAs were reported to have altered expression in ovarian cancer. Three of these miRNAs were predicted to target KLK10. We experimentally verified the predicted miR–KLK10 interaction using two independent techniques, a luciferase assay with a construct containing the KLK10 3′ untranslated region (UTR), pMIR–KLK10, and measuring KLK10 protein levels after transfection with miRNA.

**Results:**

When we co-transfected cells with pMIR–KLK10 and either let-7f, miR-224, or mR-516a, we saw decreased luciferase signal, suggesting that these miRNAs can target KLK10. We then examined the effect of these three miRNAs on KLK10 protein expression and cell growth. Transfection of all miRNAs, let-7f, miR-224, and miR-516a led to a decrease in protein expression and cellular growth. This effect was shown to be dose dependent. The KLK10 protein levels were partially restored by co-transfecting let-7f and its inhibitor. In addition, there was a slight decrease in KLK10 mRNA expression after transfection with let-7f.

**Conclusion:**

Our results confirm that KLKs can be targeted by more than one miRNA. Increased expression of certain miRNAs in ovarian cancer can lead to decreased KLK protein expression and subsequently have a negative effect on cell proliferation. This dose-dependent effect suggests that a ‘tweaking’ or ‘fine-tuning’ mechanism exists in which the expression of one KLK can be controlled by multiple miRNAs. These data together suggest that miRNA may be used as potential therapeutic options and further studies are required.

MicroRNAs (miRNAs) are small RNAs that do not code for proteins, but function by controlling protein expression of other genes. MiRNAs have been shown to control cell growth, differentiation, and apoptosis. Shortly after their discovery, miRNAs were found to be associated with cancer. Many reports have documented dysregulation of miRNAs in various malignancies (Calin and Croce, 2006a). Moreover, earlier studies have shown a tendency of cancer-dysregulated miRNAs to be located in cancer hotspot chromosomal regions, such as fragile sites, regions of loss of heterozygosity, amplification, or common breakpoint regions (Calin and Croce, 2006b). In human beings, most miRNAs function by imperfect complementarity to the 3′ untranslated region (UTR) of their target mRNAs. Recent evidence has also shown that miRNAs can also bind outside the 3′UTR of their targets, including the 5′UTR and the coding sequence (Lewis *et al*, 2005; Nakamoto *et al*, 2005; Miranda *et al*, 2006; Stark *et al*, 2007; Tay *et al*, 2008).

The association of kallikrein-related peptidases (KLKs) and cancer is well documented in the literature and many KLKs have been shown to be dysregulated in different malignancies (Emami and Diamandis, 2008). Several independent reports have shown that many KLKs are dysregulated in ovarian cancer. At least seven kallikrein proteins are up-regulated in ovarian cancer compared with normal ovarian tissues (Yousef *et al*, 2003b). The prognostic value of at least 11 out of 15 members of the human kallikrein family in ovarian cancer has been also published (Clements *et al*, 2004; Yousef *et al*, 2005; Prezas *et al*, 2006; Dorn *et al*, 2007). Six kallikreins, KLK4, KLK5, KLK6, KLK7, KLK10, and KLK15 (Kim *et al*, 2001; Kyriakopoulou *et al*, 2003; Shvartsman *et al*, 2003; Yousef *et al*, 2003c; Kountourakis *et al*, 2008), are markers of poor prognosis in ovarian cancer. Two kallikreins, KLK9 (Yousef *et al*, 2001) and KLK14 (Yousef *et al*, 2003a), are markers of favourable prognosis. In addition, KLK8 (Borgono *et al*, 2006; Kountourakis *et al*, 2009), KLK11 (Borgono *et al*, 2003; Shigemasa *et al*, 2004), and KLK13 (Scorilas *et al*, 2004; White *et al*, 2009) have conflicting evidence on prognosis. A recent report showed a concordant higher expression of both KLK5 and KLK7 in ovarian carcinomas, especially late-stage serous carcinomas, compared with normal ovaries and benign adenomas (Dong *et al*, 2003). As serine proteases, the most accepted hypothesis of function of KLKs in ovarian cancer is to enhance metastasis by extracellular matrix degradation. Recent reports, however, elucidated other possible functions of KLKs in controlling cell proliferation (Yousef and Diamandis, 2002).

Kallikrein-related peptidase 10 is a member of the kallikrein family that has been shown by numerous reports to be up-regulated in ovarian cancer (Luo *et al*, 2001, 2003; Bayani *et al*, 2008). The KLK10 protein was also purified from ascites fluid of ovarian cancer patients (Luo *et al*, 2006). Reports have shown diagnostic and prognostic uses of KLK10 in ovarian cancer (Shvartsman *et al*, 2003), and as such, it represents a possible therapeutic target for metastatic ovarian cancer.

Kallikrein functions are regulated at various levels, including transcription, translation, and post-translation (Yousef *et al*, 2005; Emami and Diamandis, 2007). A number of mechanisms have been proposed. Steroid hormones are reported to have a function in controlling KLK expression, but they, however, cannot be the sole factor controlling KLK expression in cancer (Yousef and Diamandis, 2002), as there are relatively short distances between adjacent kallikrein genes [as short as 1.5 kb between *KLK1* and *KLK15* (Yousef *et al*, 2000)], with no classic promoter elements or hormone response elements. Other possible regulatory mechanisms of kallikrein gene expression are hypermethylation of CpG islands and modification of the chromatin structure of the kallikrein locus. This has been reported in breast cancer and lymphoblastic leukaemia (Pampalakis and Sotiropoulou, 2006). At the protein level, mechanisms for controlling serine protease activity include producing kallikreins in an inactive ‘proenzymes’ (or zymogens) that are activated only when necessary. Once activated, serine proteases are controlled by ubiquitous endogenous inhibitors. Another interesting feature is the parallel co-expression of many KLKs in physiological and pathological states (Shaw and Diamandis, 2007).

Obvious discrepancies were observed in the expression profiles of many kallikreins between the mRNA and protein levels, and a post-transcriptional control mechanism was suggested (Yousef *et al*, 2005). MiRNAs represent potential candidates for this function. This is further supported by the finding that many KLKs show parallel over-expression in ovarian cancer, raising the possibility of the existence of other mechanisms that simultaneously control expression of groups of kallikreins.

We have recently published the first manuscript of *in silico* and experimental-based analysis of the potential of KLK regulation by miRNAs (Chow *et al*, 2008). We also provided preliminary experimental validation of the miRNA target prediction analysis. In this manuscript, we further test the hypothesis that multiple miRNAs can target the same KLK transcript and provide evidence that miRNAs can have a negative effect on cell proliferation through regulating KLK10 protein expression.

## Materials and methods

### Bioinformatic analysis

#### In silico expression and comparative genomic hybridisation analyses

Dysregulated miRNAs in ovarian cancer was compiled from eight published studies (Iorio *et al*, 2007; Bearfoot *et al*, 2008; Dahiya *et al*, 2008; Nam *et al*, 2008; Taylor and Gercel-Taylor, 2008; Zhang *et al*, 2008; Boren *et al*, 2009; Resnick *et al*, 2009). Assessment of KLK protein dysregulation in ovarian cancer was compiled from the results of 17 studies (Dong *et al*, 2001; Luo *et al*, 2001, 2003; Tanimoto *et al*, 2001; Hoffman *et al*, 2002; Yousef *et al*, 2002, 2003b; Borgono *et al*, 2003; Diamandis *et al*, 2003; Ni *et al*, 2004; Scorilas *et al*, 2004; Xi *et al*, 2004; Prezas *et al*, 2006; Shan *et al*, 2007; Bayani *et al*, 2008; Shaw and Diamandis, 2008). Chromosomal aberration in ovarian cancer was extracted from the Progenetix comparative genomic hybridisation database available as of January 2009 (Baudis and Cleary, 2001). Average genetic changes from 543 cases of ovarian cancer were used for analysis.

#### Target prediction analysis

Target prediction analyses were performed using multiple algorithms, including miRanda (John *et al*, 2004), PicTar (Rajewsky and Socci, 2004), and TargetScanS (Lewis *et al*, 2003). Predictions from different programmes were compiled using the TargetCombo algorithm (Sethupathy *et al*, 2006), and the miRecords target prediction programme, which complies predictions from 11 different algorithms (http://mirecords.umn.edu/miRecords/prediction_query.php). To achieve a balanced sensitivity and specificity, only predictions by more than four programmes (using the miRecords analysis) or those identified by *Optimised Intersection (which includes both PicTar, TargetScanS)* (using TargetCombo analysis) were included in the study. Optimised intersection is suggested to provide a good compromise between sensitivity and specificity for gene prediction programmes. We used the latest versions available of each programme at the time of the analysis (January 2009).

### Total RNA extraction and quantitative RT–PCR

Total RNA extraction was performed with the mirVana extraction kit, following the manufacturer's protocol (Ambion, Austin, TX, USA). The quality of extracted RNA was assessed by electropherogram and gel analysis. Quantitative RT–PCR of miRNA was performed with the TaqMan miRNA Assay kit according to the manufacture's protocol (Applied Biosystems, Foster City, CA, USA). The miRNA transcripts of three of the miRNAs predicted to target KLK10, let-7f, miR-224, and miR-516a were first reverse-transcribed into cDNA using gene-specific primers. This was followed by real-time PCR amplification using ABI7500 Standard system and miRNA-specific probes in triplicate (Applied Biosystems). Expression values were normalised to a small nucleolar RNA, RNU44 (Applied Biosystems), which has been proven to have consistent expression levels in malignant and non-malignant tissue pairs (Nikiforova *et al*, 2008). Ct values were calculated by the ABI7500. *Δ*Ct values were calculated using the Ct values of the miRNA probes and the RNU44 for each corresponding sample.

### Luciferase assay

To experimentally validate the predicted miRNA–KLK10 interactions, we transfected the pMIR-report luciferase construct (Applied Biosystems) with miRNAs in cell line models. The predicted target region of the KLK10 3′UTR (GenBank accession # NM_002776) was cloned into the vector using the primer sequences: forward 5′ cccactagtcctggatcaataaagtcatacgc 3′, and reverse 5′ tcatgtaaggcttaacacagtggaagcttccc 3′ (Operon, Huntsville, AL, USA) and called pMIR–KLK10.

Transfection of each miRNA was carried out according to the siPORT NeoFX transfection protocol (Ambion). Transfection agent/RNA molecules complex formation was carried out in Opti-MEM Reduced-Serum Media (Gibco, Burlington, Canada.). Cells were allowed to grow after transfection for 3 days and then lyzed using lysis buffer (Applied Biosystems). The luciferase and *β*-gal activity of the samples were assayed using the Dual-Light System (Applied Biosystems) according to the manufacture's protocol.

### KLK10 protein assay

The KLK10 protein expression was measured by enzyme-linked immunosorbent assay as described earlier (Luo *et al*, 2006; Shaw and Diamandis, 2007). The KLK10 antibody was first immobilised on a 96-well white polystyrene plate (200 ng per well) by incubating in coating buffer [50 mmol l^–1^ Tris and 0.05% sodium azide (pH 7.8)] overnight at room temperature. The plate was then washed three times with washing buffer [50 mmol l^–1^ Tris, 150 mmol l^–1^ NaCl, and 0.05% Tween 20 (pH 7.8)]. The KLK10 standards or samples were added to each well (100 *μ*l per well), diluted 1 : 1 in assay buffer [50 mmol l^–1^ Tris, 6% bovine serum albumin, 10% goat IgG, 2% mouse IgG, 1% bovine IgG, 0.5 mol l^–1^ KCl, and 0.05% sodium azide (pH 7.8)], incubated for 2 h with shaking, and then washed six times, as above.

Subsequently, biotinylated polyclonal detection KLK10 antibody was diluted 2000-fold in assay buffer, was added to the plate, and incubated for 1 h. After incubation, the plate was washed as above, and alkaline phosphatase-conjugated goat anti-rabbit IgG, diluted 2000-fold in assay buffer, was applied and plates were incubated for 15 min. After washing as above, the alkaline phosphatase substrate diflunisal phosphate in substrate buffer [0.1 mol l^–1^ Tris (pH 9.1), 0.1 mol l^–1^ NaCl, and 1 mmol l^–1^ MgCl_2_] was added to each well and incubated for 10 min followed by addition of developing solution (1 mol l^–1^ Tris base, 0.4 mol l^–1^ NaOH, 2 mmol l^–1^ TbCl_3_, and 3 mmol l^–1^ EDTA) for 1 min. The resultant fluorescence was measured by time-resolved fluorometry with the envision time-resolved fluorometer (Perkin-Elmer, Waltham, USA). The assay covered a linear range of detection of 0.05–10 ng ml^–1^.

### Cell growth assay

The ovarian cancer cell line OVCAR-3 was selected for transfection of KLK10-targeting miRNA to measure the effect on both cell growth and KLK10 protein expression. Cells were grown in RPMI media (Gibco, Burlington, CA, USA) supplemented with 10% foetal bovine serum and 0.01 mg ml^–1^ bovine insulin with 5% CO2 and 37°C. The siPORT NeoFX transfection system (Ambion) was used. Cells were transfected with pre-miR hsa-let-7f, hsa-miR-224, hsa-miR-516a, and a random sequence as a control. All precursor molecules (± anti-miR antagonist molecule for each miRNA) were added at a final concentration of 30 nM. All transfections were performed at the same time point, using siPORT Neo-FX transfection reagent (Ambion), according to manufacturer's protocol. Cells from the stock flasks were trypsinised and counted. The experiment was performed in duplicate.

## Results

### *In silico* analysis of miRNA dysregulation in ovarian cancer

To elucidate the miRNA–KLK interaction in ovarian cancer, we first analysed miRNA expression in ovarian cancer *in silico*. Compiled data from eight published studies were used for analysis (see Materials and Methods section). As shown in Table 1, 99 miRNAs were documented to have altered expression in ovarian cancer; 46 up-regulated and 49 down-regulated, and 4 with conflicting dysregulation patterns in 4 independent studies. Of the 46 up-regulated miRNAs, 11 were reported in multiple studies. Of the 49 down-regulated miRNAs, 13 were reported in multiple studies. Not surprisingly, miR-21, one of the most widely studied oncogenic miRNAs, was reported to be up-regulated in ovarian cancer in five independent studies (Nam *et al*, 2008; Taylor and Gercel-Taylor, 2008; Zhang *et al*, 2008; Boren *et al*, 2009; Resnick *et al*, 2009). Mapping these miRNAs along the human genome, we identified 10 miRNA clusters (with distances <50 kb between two adjacent miRNAs) located in 1p, 1q, 5q, 9q, 11q, 12p, 14q, 19q, and 22q. Most of these clusters contain only two miRNAs. Members of the same cluster could be under the same regulatory mechanisms leading to co-dysregulation of these miRNAs, and subsequently their KLK targets, in ovarian cancer. This may explain the parallel dysregulation of many KLKs in ovarian cancer that have been reported earlier.

### *In silico* analysis of KLKs as miRNA targets in ovarian cancer

The KLK expression in ovarian cancer was assessed by compiling data from 14 published studies (see Materials and Methods section). A total of 11 KLKs (KLK4-11, 13–15) were reported to be dysregulated in ovarian cancer, mostly up-regulated. In addition to their diagnostic usage, many have potential values as prognostic markers, or predictive markers for treatment efficiency.

Target prediction analysis using three different programmes showed that 62 miRNAs that are dysregulated in ovarian cancer are predicted to target KLKs (Table 2). All KLKs, except KLK14, were predicted to be targets for ovarian cancer-dysregulated miRNAs. As reported earlier, multiple miRNAs were predicted to target the same KLK and the same miRNA was predicted to target multiple KLKs (Chow *et al*, 2008; Yousef, 2008). There were no miRNAs that were predicted to target more than five KLKs.

We then examined the presence of correlated expression patterns, that is down-regulation of the miRNA is associated with KLK protein up-regulation and vice versa. Of the 125 occurrences (Table 2), 61 (49%) of the dysregulated miRNAs correlated with KLK expression in ovarian cancer.

Nine down-regulated miRNAs that target KLKs belong to the large miRNA cluster in the long arm of chromosome 19, which contains both the KLK locus and 48 miRNAs that can possibly target KLKs, suggesting the possibility of these being co-regulated in cancer. The KLKs 7, 9 10, 11, and 13 are the KLKs that are predicted to be targets of the members of this miRNA cluster.

We then *in silico* tested the hypothesis that chromosomal alterations (gains or losses) could be, at least partially, responsible for miRNA dysregulation with subsequent alteration of KLK protein levels in ovarian cancer. We correlated the expression pattern of predicted KLK-targeting miRNAs that are dysregulated in ovarian cancer with reported chromosomal aberration in the same malignancy through publicly available databases (see Materials and Methods section). Our results showed that 10 up-regulated miRNAs were located in chromosomal hotspots that frequently showed chromosomal gain in ovarian cancer. In addition, 15 of the down-regulated miRNAs were located in chromosomal hotspots that frequently showed chromosomal loss in ovarian cancer, suggesting that the dysregulation of miRNAs in ovarian cancer may be partially explained by chromosomal aberrations.

### Phylogenetic analysis

Sequence comparison of miRNAs among species was performed through the University of California Santa Cruise Genome Browser (Kent *et al*, 2002), using the Vertebrate Multiz Alignment and PhastCons Conservation (multiple alignments of 28 vertebrate species and two measures of evolutionary conservation). It was found that let-7f, which has two copies in the human genome (on chromosomes 9 and X) is highly conserved among mammalian species, as was miR-224. However, miR-516a, which has two copies on chromosome 19, is not conserved among mammalian species. Many miRNAs within the human genome, particularly the earlier discovered miRNAs are conserved among species. Phylogenetic conservation implies an important conserved function during evolution.

### miRNA target validation: effect of miRNAs on KLK10

**Figure 1:**
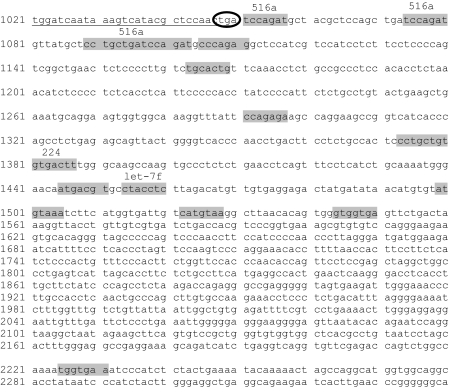
Partial sequence of the 3′UTR region for KLK10 (transcript variant 1). Nucleotide positions refer to GenBank accession # NM_002776. The position of the stop codon is circled. The miRNA predicted target sites are highlighted in grey with the targeting miRNA indicated above each site.

**Figure 2:**
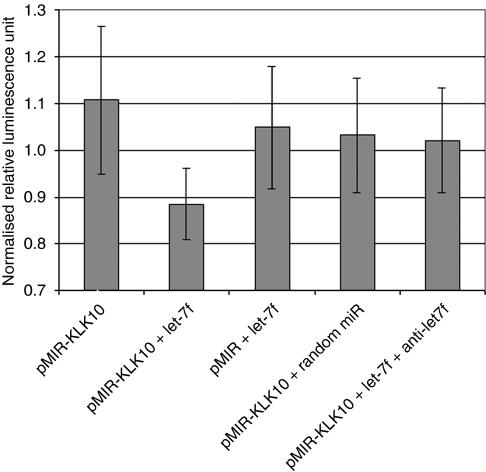
Effect of transfection of hsa-let-7f with pMIR–KLK10 on luciferase activity. Cells were transfected with either pMIR–KLK10, pMIR–KLK10 and has-let-7f precursor molecule, empty vector and has-let-7f precursor molecule, pMIR–KLK10 and a random miR sequence, or the pMIR–KLK10 with has-let-7f and the let-7f inhibitor. There was a decrease in luciferase signal when cells were transfected with pMIR–KLK10 and let-7f. The signal was partially restored when cells were co-transfected with pMIR–KLK10, let-7f, and anti-let-7f. Luciferase signals were normalised with *β*-gal.

The KLK10, which is documented to be up-regulated in ovarian cancer by many independent reports, was chosen for experimental validation of *in silico* target prediction analyses. We tested the hypothesis that multiple miRNAs can target the same KLK, and examined the correlation between KLK protein suppression by miRNAs on tumour cell proliferation. Prediction analysis from different programmes showed that the 3′UTR of KLK10 mRNA can be targeted by multiple miRNAs, including let-7f, miR-224, and miR-516a (Figure 1). These miRNAs are to be related to oncogenesis and were also reported dysregulated in ovarian cancer (Table 2). We experimentally validated the KLK10–miR interactions using the pMIR–KLK10 construct. We chose to use the OVCAR-3 ovarian cancer cell line as it has low endogenous expression of miR-224 and no expression of let-7f (data not shown). pMIR–KLK10 was transfected either alone or in combination with the precursor molecule of let-7f in OVCAR-3 cells. Successful transfection was confirmed by qRT–PCR of the corresponding miRNAs. Figure 2 shows that co-transfection of let-7f and pMIR–KLK10 led to a significant suppression of the luciferase signal. Such suppression in luciferase signal was not evident when co-transfecting a random miRNA sequence control and pMIR–KLK10 or let-7f and empty luciferase construct. The luciferase signal was partially restored when co-transfecting let-7f and its inhibitor sequence. These data confirm that let-7f specifically targets the KLK10 3′UTR.

**Figure 3:**
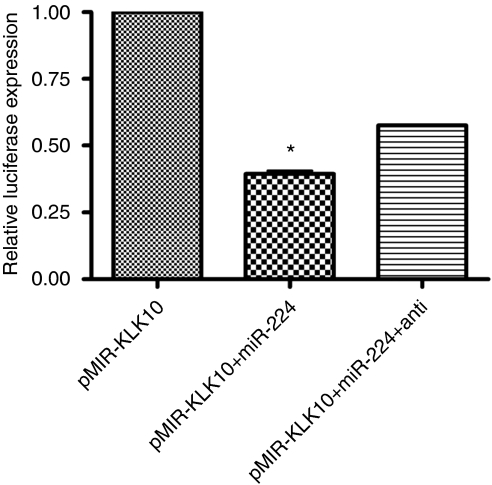
Effect of transfection of miR-224 with pMIR–KLK10 on luciferase activity. Luciferase activity was significantly decreased in OVCAR-3 cells when miR-224 was co-transfected with pMIR–KLK10 (*P*<0.001). Luciferase signal was partially restored when cells were co-transfected with pMIR–KLK10 and miR-224 and its inhibitor anti-miR-224. ^*^P<0.001.

To further validate miRNA target prediction and to confirm that multiple miRNAs can target one KLK, we preformed the same luciferase reporter assay using miR-224 in OVCAR-3 cells (Figure 3). We observed a significant decrease in luciferase signal when we co-transfected pMIR–KLK10 and miR-224 (*P*<0.001). When we transfected pMIR–KLK10, mir-224 and anti-miR-224, there was partial restoration of luciferase signal. These data together confirm that let-7f and miR-224 can both target the 3′UTR of KLK10.

**Figure 4:**
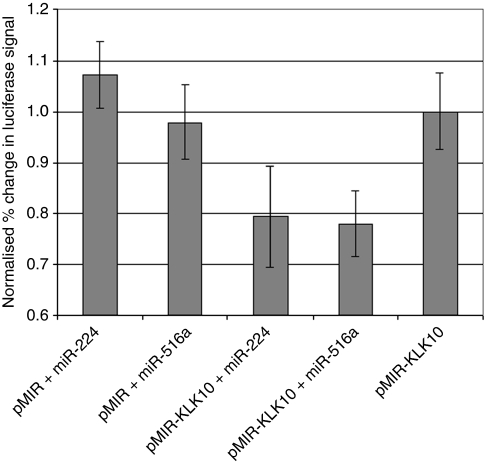
Effect of transfection of miR-224 and miR-516a on luciferase activity as measured with the pMIR–KLK10. Cells were co-transfected with the empty vector, pMIR and hsa-miR-224 precursor molecule, the empty vector, pMIR and hsa-miR-516a precursor molecule, the cloned vector, pMIR–KLK10 and hsa-miR-224 precursor molecule, pMIR–KLK10 and hsa-miR-516a precursor molecule, or pMIR–KLK10 only. There was a decrease in luciferase activity when cells were co-transfected with pMIR–KLK10 and either miR-224 or miR-516a.

We further validated our results by co-transfecting pMIR–KLK10 with miR-224 and miR-516a in the LNCaP prostate cancer cell line (Figure 4). Transfection of either of these miRNAs with pMIR–KLK10 decreased luciferase signal confirming that miR-224 and miR-516a can target KLK10. Co-transfection of either of the miRNAs with an empty vector did not result in depression of luciferase signal.

### Correlation between decreased KLK10 expression, miRNA over-expression, and cell proliferation

**Figure 5:**
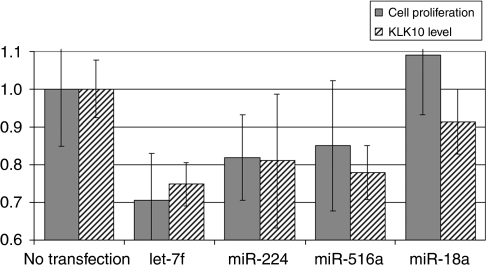
A bar graph showing the effect of miRNA transfection on KLK10 protein level (dashed bars) and cell proliferation (grey bars) in ovarian cancer cells. The transfection of any of the three miRNAs, let-7f, miR-224, and miR-516a decreased both KLK10 protein levels and cellular proliferation in the OVCAR-5 ovarian cancer cell line when compared with untransfected controls. The transfection of miR-18 was used as positive control, as it is known to have an oncogenic effect on cellular proliferation.

**Figure 6:**
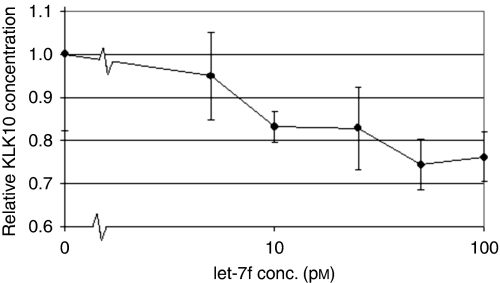
A dose curve analysis of the effect of transfected let-7f concentration on KLK10 protein level. Transfection of let-7f was found to inhibit KLK10 protein production in a dose-dependent manner.

To test the downstream biological effect of miRNAs on KLK10, we measured ovarian cancer cell proliferation. We transfected the ovarian cancer cell line OVCAR-5 with let-7f, miR-224, and miR-516a (Figure 5). The miR-18a was included as a positive control as it has been shown to have an oncogenic effect on cellular proliferation. Transfection of any of the three miRNAs decreased both KLK10 protein expression levels and the rate of cellular proliferation when compared with the untransfected control cells. Cells transfected with let-7f showed the greatest suppression in both KLK10 expression and proliferation when compared with the other miRNAs. The cells transfected with miR-224 or miR-516a showed a similar trend with decreased KLK10 expression and cell growth. We further examined whether let-7f could affect KLK10 expression in dose-dependent manner (Figure 6). We found that the suppressor effect of let-7f miRNA on KLK10 protein expression was in fact dose dependent, with lower KLK10 protein levels associated with higher miRNA transfection concentrations.

**Figure 7:**
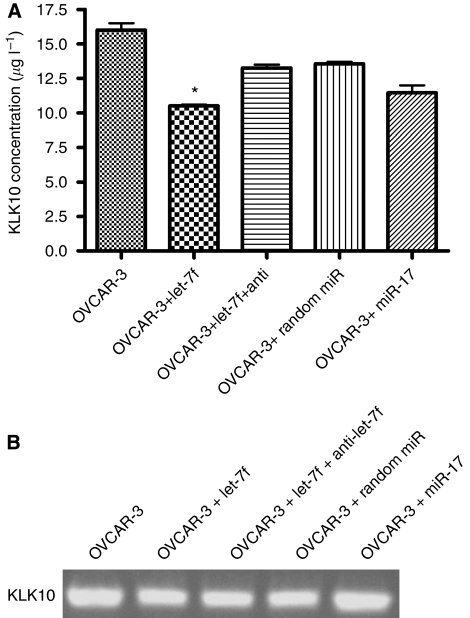
The effect of let-7f on KLK10 protein and mRNA expression in the OVCAR-3 cell line. (**A**) KLK10 protein expression was significantly decreased in cells transfected with let-7f (*P*<0.01). Protein expression was partially restored when cells were co-transfected with let-7f and its inhibitor anti-let-7f. There was a slight decrease in KLK10 protein expression when cells were transfected with a random sequence miR and miR-17. (**B**) There was a slight decrease in OVCAR-3 KLK10 mRNA expression when compared with cells transfected with either let-7f, let-7f and anti-let-7f, random sequence miR, or miR-17. ^*^, *P*<0.01.

We also compared KLK10 expression, at both the mRNA and protein levels, before and after transfection with let-7f in OVCAR-3 cells, another ovarian caner cell line. We chose to use miR-17 as a negative control, as it has partial sequence homology to let-7f and is not predicted to target KLK10. When we transfected OVCAR-3 cells with let-7f alone, let-7f and its inhibitor, a random miR sequence, and miR-17, we found that KLK10 protein expression was significantly decreased in cells transfected with let-7f, but not controls (*P*<0.001; Figure 7A). Transfection with let-7f and anti-let-7f partially restored expression levels, indicating the specificity of the miR–KLK10 interaction. There was a slight decrease in KLK10 protein expression levels when cells were transfected with the random miR and miR-17 that may be attributed to an indirect effect by consequently targeting a protein known to be involved with KLK10. The KLK10 mRNA expression levels showed only slight decrease after miRNA transfection (Figure 7B). Decreased mRNA expression after miRNA transfection has been reported earlier (Lim *et al*, 2005; Yu *et al*, 2005; Nishi *et al*, 2009; Pan *et al*, 2009; Qu *et al*, 2009; Arora *et al*, 2010), and may be due to partial degradation of the KLK10 transcript or other indirect effects.

Our results suggest that over-expression of KLK10-targeting miRNAs can lead to KLK10 protein down-regulation with subsequent negative effect on tumour cell proliferation. The KLKs represent downstream targets by which miRNAs can affect ovarian cancer pathogenesis. They also show that therapeutic over-expression of miRNAs that target KLK10 can lead to a reduction in tumour cell growth and proliferation. We also showed, for the first time, the presence of fine-tuning (quantitative) KLK protein expression in ovarian cancer as the same KLK can be controlled by multiple miRNAs.

## Discussion

Epithelial ovarian cancer is the most lethal gynaecologic malignancy (Brucks, 1992). The high-mortality rate is ascribed to late diagnosis, as epithelial ovarian tumours commonly lack early warning symptoms. Furthermore, ovarian carcinomas often lack definite precursor lesions, are quite heterogeneous, and the molecular pathways underlying their progression are still elusive. Thus, many attempts have been made to predict the biology of ovarian tumours, to determine prognosis and develop new treatment strategies. In this study, however, we provide the *first evidence* of experimental validation that three different miRNAs (let-7f, miR-224, and miR-516a) can target KLK10 (using two distinct approaches) and subsequently have an effect on ovarian cancer cell proliferation.

Our findings should be, however, interpreted with caution, as the effect of let-7f (and other miRNAs) on cell proliferation can be mediated through other targeted genes. We also provide a unique association between miRNA dysregulation, KLK protein expression level, and ovarian cancer cell proliferation in a dose-dependent manner. Together with published data about potential effect of KLKs on ovarian cancer proliferation, these data strongly suggest that KLK10 is a candidate downstream target by which miRNA can affect ovarian cancer proliferation, although further experimental validation is required. Regardless of this effect being mediated through kallikreins, we provided strong evidence to suggest the presence of a functional involvement of dysregulated miRNAs on ovarian cancer proliferation.

Dysregulation of KLKs in ovarian cancer is well established in the literature. There are several mechanisms that are hypothesised for the dysregulation of KLKs in ovarian cancer. Many reports have shown that many KLK mRNA transcripts are over-expressed in cancer (Klokk *et al*, 2007; Mange *et al*, 2008; Pettus *et al*, 2009). Hormonal control is another postulated mechanism (Yousef and Diamandis, 1999; Shaw and Diamandis, 2008; Lai *et al*, 2009). However, there are also reports that suggest that other regulatory mechanism exists for KLK expression in cancers (Shan *et al*, 2007). Our findings highlight the possibility that miRNAs represent a potential regulatory mechanism for controlling kallikrein expression at the post-transcriptional level. The finding that many miRNAs can target the same KLK and a single miRNA can target more than one kallikrein suggest that miRNAs can exert a ‘quantitative’ control of kallikreins by using multiple targeting sites in the kallikrein mRNA.

Many of the miRNAs that were shown to be dysregulated in ovarian cancer and able to target KLKs were also shown to be dysregulated in other malignancies. For example, hsa-let-7f was found to be significantly up-regulated in breast cancer (Yan *et al*, 2008), and hsa-miR-224 was found to be significantly up-regulated in prostate and thyroid cancer (Prueitt *et al*, 2008; Nikiforova *et al*, 2008). This might point out the presence of ‘common’ pathways that are used by cancers in different organ tissues. It should be also noted that these miRNAs have other predicted and experimentally validated targets. For example, miR-224 is up-regulated in highly invasion pancreatic ductal adenocarcinoma, and it is correlated with the down-regulation of CD40, which it is predicted to target (Mees *et al*, 2009). MiR-224 is also predicted to target apoptosis inhibitor-5 in hepatocellular carcinoma; it is found to be significantly up-regulated in hepatocellular carcinoma and as a result inhibits apoptosis and induces tumourigenesis (Wang *et al*, 2008). This indicates the presence of synergetic or harmonised effects of miRNAs in cancer initiation and progression through targeting multiple pathways.

The miRNA-based cancer gene therapy offers the theoretical appeal of targeting multiple gene networks that are controlled by a single, aberrantly expressed miRNA (Tong and Nemunaitis, 2008). Reconstitution of tumour-suppressor miRNAs or sequence-specific knockdown of oncogenic miRNAs has produced favourable anti-tumour outcomes in experimental models and are now being tested for therapeutic applications in different cancers (Garzon *et al*, 2006).

Our results show the potential usage of miRNAs or their synthetic counterparts, small-interfering RNAs (siRNAs), to alter KLK expression in cancer for therapeutic purposes. Reports have shown that siRNA and RNAi were able to knockdown kallikrein gene and protein expression (Bando *et al*, 2006; Klokk *et al*, 2007; Shinoda *et al*, 2007). It should be noted, however, that the endogenous or exogenous siRNAs and synthetic RNAi do not function exactly similar to miRNAs (Yousef, 2008). Endogenous siRNAs are derived from a double-stranded DNA precursors, and have exact complementarity with their target sequence, whereas miRNA form a local hairpin structure and the mature form is a single-stranded molecule (Ambros *et al*, 2003). The negative regulatory effect of miRNAs in animals occurs by imperfect complementarity to the 3′UTR of the target, causing suppression of protein translation through an RNA-induced silencing complex pathway (Esquela-Kerscher and Slack, 2006).

Another potential interesting clinical application is the use of KLK proteins as markers for miRNA treatment efficiency in the future. Other means of inhibiting or inactivating KLK proteins in cancer cells represent another alternative to block miRNA pathways in ovarian cancer. Blocking both KLKs and miRNAs might augment the therapeutic effect.

Our target prediction analysis highlights the need for high throughput target validation approaches. At the moment, there is no ‘gold standard’ programme for miRNA target prediction analysis, and the overall sensitivity rate is 65–68%. Our *in silico* analysis showed a number of miRNA clusters within the genome. The largest cluster is on chromosome 19, very close to the kallikrein gene cluster. The two clusters can be simultaneous targets to a control mechanism that affects this region, for example locus control region or chromosomal insertion or deletions.

Our results were based on target prediction of the ‘classic’ form of KLK genes. An added layer of complexity that should be considered is the presence of many splice variants for kallikrein genes, with some of them differ in their 3′UTR (Kurlender *et al*, 2005). It is also important to examine whether these splice variations can affect KLK protein expression by altering miRNA-binding sites.

In conclusion, our combined bioinformatics and experimental approach provides evidence that KLK10 is a downstream target for multiple miRNAs in ovarian cancer, with a positive proliferative effect on cancer cell proliferation. More detailed analyses are needed for target validation of other KLKs and exploring the possible involvement of miRNAs in controlling kallikrein gene expression in ovarian cancer pathogenesis.

## Figures and Tables

**Table 1:** A list of all published miRNAs that were documented to be dysregulated in ovarian cancer^a^

**miRNA**	**# up**	**# down**	**miRNA**	**# up**	**# down**
hsa-let-7a		1	hsa-miR-21	5	
hsa-let-7b		1	hsa-miR-210	2	
hsa-let-7d		1	hsa-miR-213	2	
hsa-let-7e	1		hsa-miR-214	2	1
hsa-let-7f		1	hsa-miR-222		1
hsa-let-7i	1		hsa-miR-224		1
hsa-miR-100		2	hsa-miR-23a	1	
hsa-miR-101-1		1	hsa-miR-23b	2	
hsa-miR-103	1		hsa-miR-26a	1	1
hsa-miR-104		1	hsa-miR-26b	1	
hsa-miR-106a		1	hsa-miR-27a	1	
hsa-miR-10b		2	hsa-miR-299		1
hsa-miR-122a	1		hsa-miR-29a	1	1
hsa-miR-124a		1	hsa-miR-29c		1
hsa-miR-125a		2	hsa-miR-302c		1
hsa-miR-125b		4	hsa-miR-323		1
hsa-miR-126	2		hsa-miR-330		1
hsa-miR-127		2	hsa-miR-331		2
hsa-miR-128b	1		hsa-miR-337		1
hsa-miR-132		1	hsa-miR-339		1
hsa-miR-134		1	hsa-miR-340	1	
hsa-miR-136	1		hsa-miR-34a	1	
hsa-miR-140-5p		2	hsa-miR-34b	1	
hsa-miR-141	3		hsa-miR-34c	1	
hsa-miR-142-5p		1	hsa-miR-368		1
hsa-miR-143		2	hsa-miR-370		1
hsa-miR-145		4	hsa-miR-371	1	
hsa-miR-149	1		hsa-miR-376a		1
hsa-miR-150		1	hsa-miR-376b		1
hsa-miR-152		1	hsa-miR-377		1
hsa-miR-154		1	hsa-miR-381	1	
hsa-miR-155		2	hsa-miR-410		1
hsa-miR-16	1		hsa-miR-424		2
hsa-miR-181a	1		hsa-miR-431	1	
hsa-miR-181b	1		hsa-miR-432		1
hsa-miR-182	1		hsa-miR-495		1
hsa-miR-184		1	has-miR-502	1	
hsa-miR-185		1	hsa-miR-514	1	
hsa-miR-191	1		hsa-miR-515-5p	1	
hsa-miR-196a	1		hsa-miR-518c		1
hsa-miR-199a		2	hsa-miR-518c^a^	1	
hsa-miR-200a	3		hsa-miR-520f	1	
hsa-miR-200b	2		hsa-miR-9	1	
hsa-miR-200c	4		hsa-miR-92	1	
hsa-miR-202	1		hsa-miR-93	2	
hsa-miR-203	2		hsa-miR-98	1	
hsa-miR-204		1	hsa-miR-99a		2
hsa-miR-205	1		hsa-miR-99b	1	1
has-miR-206	1		has-miR-516a		1
Has-miR-20a	1				

Abbreviation: miRNA=microRNA.

a Data is compiled from eight published studies (see Materials and Methods section).

**Table 2:** miRNAs dysregulated in ovarian cancer and their predicted KLK targets

**KLK**																
**miRNA**	**1**	**2**	**3**	**4**	**5**	**6**	**7**	**8**	**9**	**10**	**11**	**12**	**13**	**14**	**15**	**Total**
hsa-let-7a						√				√						2
hsa-let-7b	√					√				√						3
hsa-let-7d						√										1
hsa-let-7e						√										1
hsa-let-7f	√					√										2
hsa-let-7i						√										1
hsa-miR-103		√			√											2
hsa-miR-106a					√		√									2
hsa-miR-10b		√					√									2
hsa-miR-122	√	√			√					√						4
hsa-miR-125b					√											1
hsa-miR-132		√														1
hsa-miR-140-5p	√					√			√	√						4
hsa-miR-141													√			1
hsa-miR-142-5p		√														1
hsa-miR-143	√	√			√					√			√			5
hsa-miR-145							√									1
hsa-miR-149	√		√							√						3
hsa-miR-152			√													1
hsa-miR-181a							√						√			2
hsa-miR-181b							√						√			2
hsa-miR-196a								√								1
hsa-miR-199a-5p		√					√									2
hsa-miR-200a													√			1
hsa-miR-200b							√									1
hsa-miR-200c							√									1
hsa-miR-204		√														1
hsa-miR-205		√										√				2
hsa-miR-206	√									√			√			3
hsa-miR-20a					√		√									2
hsa-miR-21		√														1
hsa-miR-210							√									1
hsa-miR-214	√	√			√					√						4
hsa-miR-223		√													√	2
hsa-miR-224	√									√					√	3
hsa-miR-26a		√					√									2
hsa-miR-26b		√					√									2
hsa-miR-302c		√														1
hsa-miR-323-5p							√								√	2
hsa-miR-330-5p		√		√	√		√		√							5
hsa-miR-331-5p		√										√				2
hsa-miR-337-3p							√									1
hsa-miR-339-5p		√					√									2
hsa-miR-340		√					√									2
hsa-miR-34a													√			1
hsa-miR-34b							√									1
hsa-miR-34c-5p													√			1
hsa-miR-370		√														1
hsa-miR-371-5p		√					√									2
hsa-miR-376a							√		√				√			3
hsa-miR-376b							√		√				√			3
hsa-miR-377	√	√								√						3
hsa-miR-431		√							√							2
hsa-miR-432	√	√	√							√						4
hsa-miR-495		√									√					2
hsa-miR-502-5p		√	√					√	√							4
hsa-miR-515-5p		√					√									2
hsa-miR-516a										√						1
hsa-miR-520f			√					√								2
hsa-miR-9		√									√					3
hsa-miR-93					√		√									2
hsa-miR-98						√										1
*Total*	*11*	*28*	*5*	*1*	*9*	*8*	*23*	*3*	*6*	*13*	*2*	*2*	*10*	*0*	*4*	*125*

Abbreviation: miRNA=microRNA; KLK=kallikrein-related peptidase.
